# Perioperative Venous Thromboembolism in Ulcerative Colitis: A Multicenter Prospective Study in Japan

**DOI:** 10.1093/crocol/otab024

**Published:** 2021-05-23

**Authors:** Michio Itabashi, Hiroki Ikeuchi, Hideaki Kimura, Kohei Fukushima, Hisao Fujii, Riichiro Nezu, Kitaro Futami, Akira Sugita, Yasuo Suzuki, Tadakazu Hisamatsu

**Affiliations:** 1 Department of Surgery, Institute of Gastroenterology, Tokyo Women’s Medical University Hospital, Tokyo, Japan; 2 Department of Inflammatory Bowel Disease Surgery, Hyogo College of Medicine, Hyogo, Japan; 3 Inflammatory Bowel Disease Center, Yokohama City University Medical Center, Yokohama, Japan; 4 Surgical and Molecular Pathophysiology, Tohoku University School of Medicine, Sendai, Japan; 5 Department of Surgery, Nara Medical University, Nara, Japan; 6 Nishinomiya Municipal Center Hospital, Hyogo, Japan; 7 Department of Surgery, Fukuoka University Chikushi Hospital, Fukuoka, Japan; 8 Inflammatory Bowel Disease Center, Yokohama Municipal Citizen’s Hospital, Yokohama, Japan; 9 Department of Internal Medicine, Sakura Medical Center, Toho University, Chiba, Japan; 10 Department of Gastroenterology, Kyorin University, Tokyo, Japan

**Keywords:** ulcerative colitis, venous thromboembolism, pulmonary embolism, deep vein thrombosis, inflammatory bowel disease

## Abstract

**Background:**

Recently, the prevalence of venous thromboembolism (VTE) in Asian patients with inflammatory bowel disease (IBD) is gradually increasing. IBD surgery is a well-recognized risk factor for VTE. However, there are no prospective studies about VTE after surgery for ulcerative colitis (UC) in Asia. This multicenter prospective study aimed to clarify the prevalence and risk factors for perioperative VTE in UC surgery in Japan.

**Methods:**

A total of 134 patients with UC were included from January 1, 2013 to December 31, 2014. Preoperative screening was performed in all patients. In the perioperative period, standard VTE prophylaxis based on risk assessment was administered. The prevalence of pre- and postoperative VTE, its risk factors, and mortality rates were investigated.

**Results:**

Perioperative deep vein thrombosis and pulmonary embolism were diagnosed in 15 (11.1%) and 1 patient (0.7%), respectively. All patients were asymptomatic. No surgery-related deaths were found (mortality rate 0%). Seven patients (5.2%) were diagnosed, and 8 (6.4%) during postoperative follow-up by ultrasonography or computed tomography. Forty-seven percent of VTE cases was developed preoperatively. A preoperative hospital length stay of over 5 days was a significant risk factor [*P* = 0.04; odds ratio: 8.26 (1.06–64.60)] for preoperative VTE. Postoperative deep vein thrombosis occurred in 8 of the 127 patients (6.4%). Six out of these 8 (75.0%) occurred after postoperative day 14. Perioperative blood transfusion was a significant risk factor [*P* = 0.04; odds ratio: 8.26 (1.06–64.60)] for postoperative VTE.

**Conclusion:**

A VTE-conscious perioperative management is as necessary in Asia as in Western countries.

## Introduction

The risk of deep vein thrombosis (DVT) in patients with inflammatory bowel disease (IBD) is higher than that of the general population.^1–3^ The incidence rate ratios of DVT and pulmonary embolism (PE) in patients with IBD and healthy individuals were 3.5, 95% confidence interval (CI) 2.9–4.3 and 3.3, 95% CI 2.5–4.3, respectively.^4^ Moreover, venous thromboembolism (VTE) is one of the leading causes of death in patients with IBD.^5, 6^

Risk factors for VTE in ulcerative colitis (UC) surgery, such as disease activity, age, hospitalization, and others have been reported in Western countries.^7–12^ The prevalence of VTE in UC surgery was 4.9% after elective surgery, and 8.7% after emergency surgery, which was 3.69 and 5.28 times higher when compared to those undergoing medical treatment, respectively.^13^

Asian patients are generally expected to have a low incidence of VTE due to their low body mass index and the low frequency of hyperlipidemia and cardiovascular diseases.^14, 15^ From July 2012 to June 2013, we performed a preoperative screening for general surgery in a similar preoperative screening manner at our facility.^16^ In a previous study, preoperative VTE was diagnosed in 12 out of 307 patients, (3.9%) including 91 patients with colorectal cancer and 27 patients with IBD.

Recently, the frequency of VTE in Asian patients with IBD is gradually increasing.^17–19^ However, reports from Asia are lacking, especially prospective studies, and the number of cases is not sufficient.^20^

The frequency and risk factors for perioperative VTE in Asian countries are currently unknown. This is the first prospective study of VTE in UC surgery in Asia. The purpose of this study was to clarify the prevalence and risk factors for perioperative VTE in UC surgery.

## Materials and Methods

This was a Japanese multicenter prospective study. From January 1, 2013 to December 31, 2014, patients with UC were recruited at participating institutions to take part in this study. Patients younger than 20 years of age at the time of surgery, those with previously diagnosed VTE, and patients on anticoagulant therapy for other diseases were excluded. The observation period of this study was from the date of admission to the date of discharge.

We examined the patients’ d-dimer levels preoperatively and on postoperative days (PODs) 3,7, and 14. Patients with a positive d-dimer assay underwent venous ultrasonography (US) or enhanced multi-detector helical computed tomography (MDCT) of the lower limbs. All patients received a comprehensive risk assessment before surgery. In the perioperative period, standard VTE prophylaxis based on risk assessment was performed in each institution. All patients were divided into 4 risk levels: very low risk, low risk, moderate risk, and high risk, using the Caprini score.^21^

Graduated compression stockings and intermittent pneumatic compression were used in all patients from just before the surgery to one day after surgery. Chemoprophylaxis was administered in high-risk patients according to the 2009 Japanese Circulation Society guidelines.^22^

We examined blood biochemistry on PODs 3, 7, and 14. US was performed on day 7 after surgery. If there were symptoms suggestive of VTE or if the d-dimer was above the reference level, US or MDCT was performed.

Prevalence of pre- and postoperative DVT, its risk factors, and mortality rates were investigated. UC disease severity was classified into mild, moderate, and severe.^23^

In the risk factor analysis, each factor was subjected to univariate analysis, and significant factors were included in multivariate analysis. *P* values less than 0.05 were determined to be significant. All analyses were performed using JMP Pro version 14 (SAS Institute, Cary, NC).

## Ethical Considerations

This prospective multicenter study conducted in Japan was approved by the institutional review board of Tokyo Women’s Medical University (approval number: 130207). In addition, ethical approval was obtained from each participating institution. Informed consent was obtained from all patients before enrollment.

## Results

**Figure 1.**
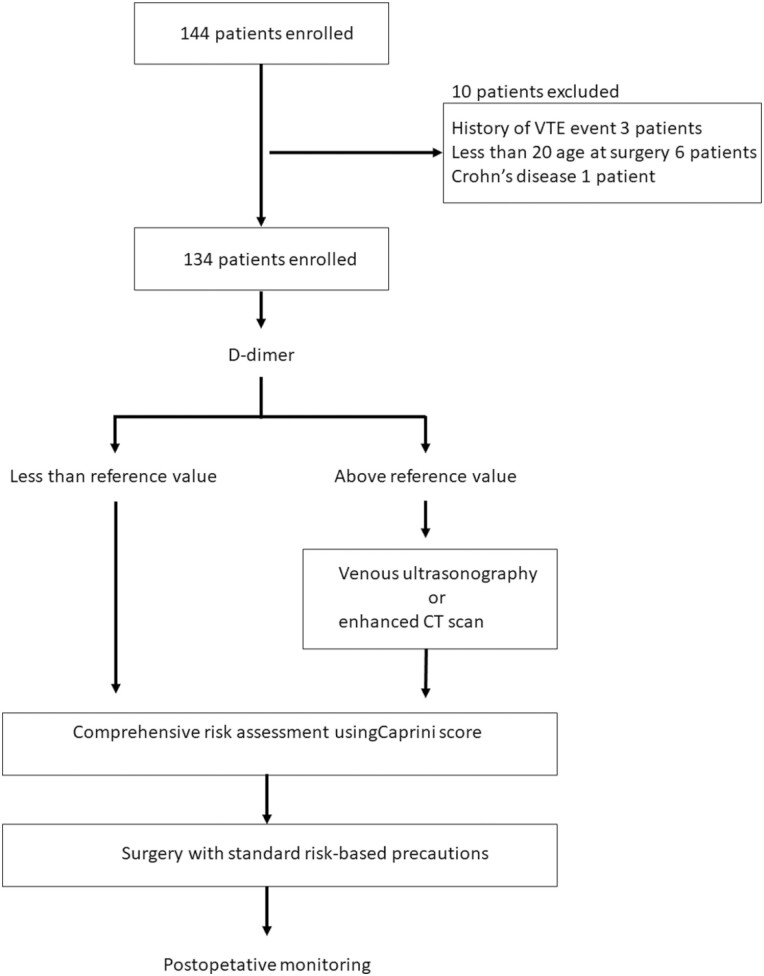
Flow diagram of patients enrolled in this study.

A total of 144 patients with UC were enrolled during the study period. Ten patients were excluded (histories of VTE event, 3 patients; younger than age 20 at surgery, 6 patients; and Crohn’s disease, 1 patient); 134 patients were therefore included in the analysis (Fig. 1). In 51 patients (37.8%), d-dimer was higher than the reference value and therefore, these patients further underwent either a US or MDCT.

Restorative proctocolectomy, subtotal colectomy, and total proctocolectomy were performed in 101 (75.4%), 24 (17.9%), and 9 (6.7%) patients, respectively. Among patients who underwent restorative proctocolectomy, 41 patients received stapled anastomosis and 60 patients received hand-sewn anastomosis. Laparoscopic procedure was performed in 5 patients (restorative proctocolectomy, 4 patients; total proctocolectomy, 1 patient). Postoperative chemoprophylaxis was given to all patients with preoperatively diagnosed VTE. In patients without VTE, chemoprophylaxis was administered in 15 out of the 119 patients (12.6%) at the attending physicians’ discretion. These patients received antithrombotic prophylaxis (Enoxaparin sodium) for 7 days starting on the morning of POD 1. Enoxaparin was injected subcutaneously at 2000 IU once every 12 hours twice a day. There are no complications due to mechanical prophylaxis and chemoprophylaxis.

Perioperative DVT was diagnosed in 15 out of the 134 patients (11.1%) without any symptoms. US and MDCT were performed in 13 and 3 patients, respectively. No surgery-related deaths were found (mortality rate 0%).

Seven patients (5.2%) were diagnosed by preoperative screening and 8 (6.4%) were diagnosed using US during the postoperative follow-up.

**Figure 2.**
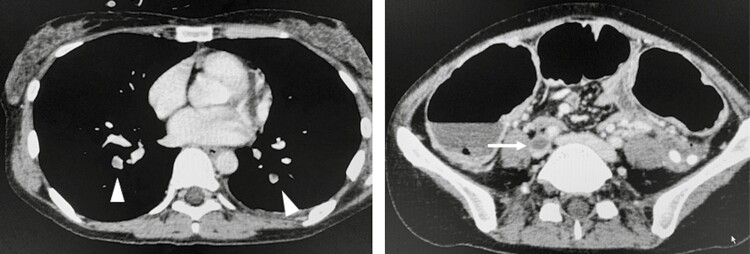
A 39-year-old woman who underwent emergency surgery for toxic megacolon. Contrast-enhanced computed tomography revealed pulmonary embolism (enhanced CT diagnosed pulmonary embolisms (denoted by △) and inferior vena cava thrombosis (denoted by →).

Preoperatively, VTEs had already developed in 47% (7 out of 15 patients) of VTE cases. DVTs were present in 7 patients (lower legs 7, inferior vena cava 1). PE was diagnosed in 1 out of the 7 patients (14.3%) preoperatively using MDCT. In one case of emergency surgery for toxic megacolon, MDCT performed for preoperative screening diagnosed an inferior vena cava thrombosis and PE (Fig. 2).

**Figure 3.**
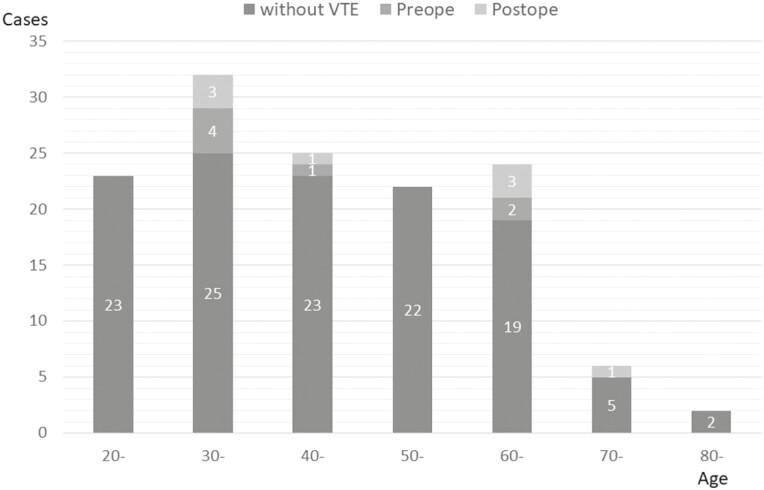
Frequency of perioperative VTE by age.

Sex, hyperlipidemia, disease severity, hospital stay before surgery, preoperative insertion of the central venous catheter, Caprini score, albumin, and d-dimer levels were found to be significant factors in the univariate analysis. Age at surgery and corticosteroid administration were not significant factors (Fig. 3). Therefore, only a hospital stay of over 5 days was a significant risk factor [*P* = 0.04; odds ratio (OR): 8.25 (1.06–64.42)] for preoperative VTE in the multivariate analysis (Table 1).

**Table 1.** Risk factors for preoperative VTE

Factors	Without VTE, N = 127	With VTE, N = 7	Univariate analysis	Multivariate analysis	
				*P*	OR (95% CI)
Age at surgery, mean ± SD, years	45.7 ± 15.6	47.1 ± 14.4	0.79		
Sex, M: F, n (%)	86 (68): 41 (32)	2 (29): 5 (71)	**0.03**	0.13	5.57 (0.60–51.83)
Body mass index, mean ± SD, kg/m^2^	19.9 ± 3.5	19.6 ± 4.4	0.64		
Smoking, no: yes: unknown, n (%)	114 (90): 9 (7): 4 (3)	6 (86): 1 (14): 0 (0)	0.71		
Drinking, no: sometimes: yes: unknown, n (%)	94 (74): 19 (15): 9 (7): 5 (4)	6 (86): 0 (0): 1 (14): 0 (0)	0.59		
Comorbidity, no: yes, n (%)	102 (80): 25 (20)	4 (57): 3 (43)	0.14		
Hypertension, no: yes: unknown, n (%)	120 (94): 7 (6): 0 (0)	5 (72): 1 (14): 1 (14)	0.26		
Hyperlipidemia, no: yes: unknown, n (%)	124 (98): 3 (2): 0 (0)	5 (72): 1 (14): 1 (14)	**0.05**	0.13	10.87 (0.48–243.95)
Diabetes mellitus, no: yes: unknown, n (%)	119 (94): 8 (6): 0 (0)	6 (86): 0 (0): 1 (14)	0.52		
Chronic kidney disease, no: yes: unknown, n (%)	126 (99): 1 (1): 0 (0)	6 (86): 0 (0): 1(14)	0.90		
Ischemic heart disease, no: yes: unknown, n (%)	124 (98): 3 (2): 0 (0)	6 (86): 0 (0): 1(14)	0.86		
Duration of disease before surgery, mean ± SD, months	103.3 ± 111.0	137.4 ± 193.1	0.66		
Extension of disease, pancolitis: left-sided: proctitis, n (%)	111 (87): 15 (12): 1 (1)	7 (100): 0 (0): 0 (0)	0.60		
Disease severity, mild: moderate: severe, n (%)	31 (24): 64 (51): 32 (25)	2 (29): 1 (14): 4 (57)	0.18		
Disease severity, mild/moderate: severe, n (%)	95 (75): 32 (25)	3 (43): 4 (57)	**0.06**	0.36	4.16 (0.20–88.45)
Indication for surgery					
Refractory, cancer and/or dysplasia, severe attack, local complication, toxic megacolon, massive bleeding, perforation, n (%)	62 (49): 34 (27): 17 (13): 6 (5): 4 (3): 3 (2): 1 (1)	3 (43): 1 (14): 2 (29): 0 (0): 1 (14): 0 (0): 0 (0)	0.64		
Colitis-associated cancer, no: yes, n (%)	98 (77): 29 (23)	6 (86): 1 (14)	0.60		
Urgency of surgery, elective: emergency, n (%)	101 (80): 26 (20)	4 (57): 3 (43)	0.16		
Hospital stay before surgery, mean ± SD, days	4.9 ± 8.7	22.0 ± 38.4	**0.02**		
Hospital stay before surgery, less than 5 days: over 5 days, n (%)	113 (89): 14 (11)	4 (57): 3(43)	**0.01**	**0.04**	**8.25 (1.06–64.42)**
Performance status before surgery, 0: 1: 2, n (%)	29 (23): 69 (54): 29 (23)	2 (28): 3 (44): 2 (28)	0.84		
Prolonged bed rest over 1 week, no: yes, n (%)	117 (92): 10 (8)	6 (86): 1 (14)	0.54		
Preoperative medical treatment, no: yes, n (%)	5 (4): 122 (96)	0 (0): 7 (100)	0.59		
Mesalamine, no: yes, n (%)	15 (12): 112 (88)	0 (0): 7 (100)	0.33		
Corticosteroid, no: yes, n (%)	47 (37): 80 (63)	1 (14): 6 (86)	0.22		
Corticosteroid 20 mg or more per day, no: yes, n (%)	105 (83): 22 (17)	4 (57): 3 (43)	0.09		
Immunomodulators, no: yes, n (%)	56 (44): 71 (56)	4 (57): 3 (43)	0.50		
Anti-TNF-α, no: yes, n (%)	83 (65): 44 (34)	5 (71): 2 (29)	0.74		
Central venous catheter insertion before surgery, no: yes, n (%)	108 (85): 19 (15)	3 (43): 4 (57)	**0.004**	0.39	3.41 (0.21–55.71)
Preoperative blood transfusion, no: yes, n (%)	121 (95): 6 (5)	7 (100): 0 (0)	0.55		
Caprini score, moderate: high risk, n (%)	78 (61): 49 (39)	1 (14): 6 (86)	**0.01**	0.24	6.97 (0.27–179.36)
Preoperative blood biochemistry findings					
Hemoglobin, mean ± SD, g/dL	11.8 ± 2.3	10.9 ± 2.7	0.32		
Total protein, mean ± SD, g/dL	6.7 ± 1.0	5.9 ± 0.8	0.01		
Albumin, mean ± SD, g/dL	3.6 ± 0.9	2.7 ± 0.8	**0.01**	0.56	0.60 (0.09–3.48)
d-dimer, below: above reference value, n (%)	82 (65): 45 (35)	1 (14): 6 (86)	**0.01**	0.56	2.34 (0.13–44.32)

**Figure 4.**
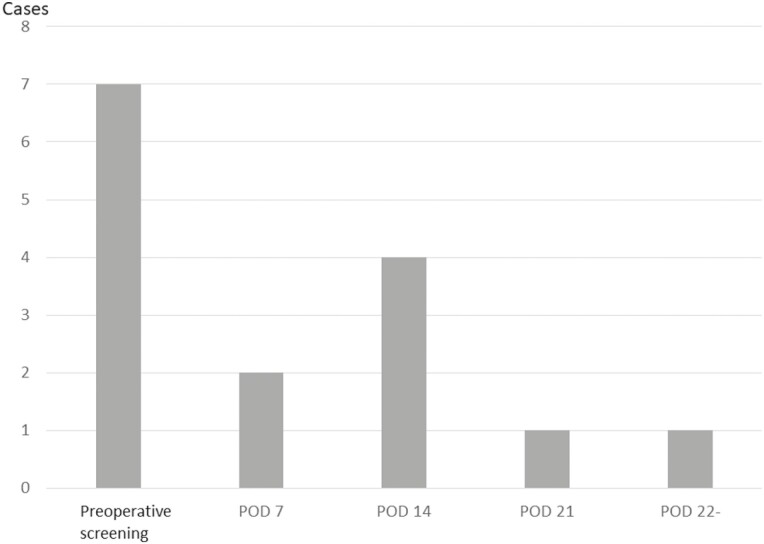
Period of VTE diagnosis.

The average length of hospital stay after surgery was 21.5 ± 12.1 days. Postoperative DVTs were diagnosed using US in 8 out of the 127 patients (6.4%). There were no cases of PE and no deaths occurred. All these patients were asymptomatic and had DVT in the lower legs. Postoperative VTE occurred in 6 out of these 8 patients (75.0%) on POD 14 or after (Fig. 4). Hyperlipidemia, ischemic heart disease, corticosteroid administration, perioperative blood transfusion, and albumin level at POD 3 were found to be significant factors associated with postoperative VTE in univariate analysis. However, age at surgery was not a significant factor (Fig. 2). Perioperative blood transfusion was a significant risk factor [*P* = 0.03; OR: 13.81 (1.22–288.58)] for postoperative VTE in the multivariate analysis (Table 2).

**Table 2.** Risk factors for postoperative VTE.

Factors	Without VTE, N = 119	With VTE, N = 8	Univariate analysis	Multivariate analysis	
				*P*	OR (95% CI)
Age at surgery, mean ± SD, years	45.2 ± 15.5	53.1 ± 16.8	0.20		
Sex, M: F, n (%)	80 (67): 39 (33)	6 (75): 2 (25)	0.64		
Body mass index, mean ± SD, kg/m^2^	19.9 ± 3.3	19.9 ± 6.0	0.60		
Smoking, no: yes: unknown, n (%)	107 (90): 8 (7): 4 (3)	7 (88): 1 (12): 0 (0)	0.73		
Drinking, no: sometimes: yes: unknown, n (%)	88 (74): 19 (16): 7 (6): 5 (4)	6 (75): 0 (0): 2 (25): 0 (0)	0.13		
Comorbidity, no: yes, n (%)	96 (81): 23 (19)	6 (75): 2 (25)	0.69		
Hypertension, no: yes: unknown, n (%)	113 (95): 6 5)	7 (88): 1 (12)	0.37		
Hyperlipidemia, no: yes: unknown, n (%)	117 (98): 2 (2)	7 (88): 1 (12)	**0.05**	0.74	2.16 (0.01–92.90)
Diabetes mellitus, no: yes: unknown, n (%)	111 (93): 8 (7)	8 (100): 0 (0)	0.44		
Chronic kidney disease, no: yes: unknown, n (%)	118 (99): 1 (1)	8 (100): 0 (0)	0.87		
Ischemic heart disease, no: yes: unknown, n (%)	118 (99): 1 (1)	6 (75): 2 (25)	**0.01**	0.81	4.45 (0.03–394.53)
Duration of disease before surgery, mean ± SD, months	100.7 ± 111.0	137.6 ± 112.3	0.20		
Extension of disease, pancolitis: left-sided: proctitis, n (%)	104 (87): 14 (12): 1 (1)	7 (87): 1 (13): 0 (0)	0.96		
Disease severity, mild: moderate: severe, n (%)	31 (26): 58 (49): 30 (25)	0 (0): 6 (75): 2 (25)	0.35		
Disease severity, mild/moderate: severe, n (%)	89 (75): 30 (25)	6 (75): 2 (25)	0.98		
Indication for surgery					
Refractory, cancer and/ or dysplasia, severe attack, local complication, toxic megacolon, massive bleeding, perforation, n (%)	56 (47): 33 (28): 16 (13): 6 (5): 4 (3): 3 (3): 1 (1)	6 (75): 1 (13): 1 (13): 0 (0): 0 (0): 0 (0): 0(0)	0.83		
Colitis-associated cancer, no: yes, n (%)	91 (76): 28 (24)	7 (88): 1 (12)	0.47		
Urgency of surgery, elective: emergency, n (%)	94 (79): 25 (21)	7 (88): 1 (12)	0.90		
Hospital stay before surgery, mean ± SD, day	5.0 ± 8.9	1.8 ± 1.4	0.18		
Hospital stay before surgery, less than 5: over 5 days, n (%)	98 (82): 21 (18)	8 (100): 0 (0)	0.19		
Performance status before surgery, 0: 1: 2, n (%)	28 (24): 64 (54): 27 (22)	1 (12): 5 (63): 2 (25)	0.77		
Prolonged bed rest over one week, no: yes, n (%)	110 (92): 9 (8)	7 (88): 1 (12)	0.61		
Preoperative medical treatment, no: yes, n (%)	5 (4): 114 (96)	0 (0): 8 (100)	0.55		
Mesalamine, no: yes, n (%)	15 (13): 104 (87)	0 (0): 8 (100)	0.28		
Corticosteroid, no: yes, n (%)	47 (39): 72 (61)	0 (0): 8 (100)	**0.03**	0.06	15330812 (0.01–1.04)
Corticosteroid 20 mg or more per day, no: yes, n (%)	98 (82): 21 (18)	7 (88): 1 (12)	0.70		
Immunomodulators, no: yes, n (%)	53 (45): 66 (55)	3 (38): 5 (42)	0.70		
Anti-TNF-α, no: yes, n (%)	78 (66): 41 (34)	5 (63): 3 (37)	0.86		
Central venous catheter insertion before surgery, no: yes, n (%)	100 (84): 19 (16)	8 (100): 0 (0)	0.22		
Perioperative blood transfusion, no: yes, n (%)	107 (90): 12 (10)	5 (63): 3 (37)	**0.02**	**0.03**	**13.81 (1.22–288.58)**
Caprini score, moderate: high risk, n (%)	74 (62): 45 (38)	4 (50): 4 (50)	0.49		
Postoperative chemoprophylaxis	104 (87): 15 (13)	8 (100): 0 (0)	0.28		
Postoperative complications, 0 or Clavien I: Clavien II/III/IV, n (%)	83 (70): 36 (30)	5 (63): 3 (37)	0.67		
Preoperative blood biochemistry					
Hemoglobin, mean ± SD, g/dL	11.9 ± 2.3	11.3 ± 2.0	0.39		
Total protein, mean ± SD, g/dL	6.8 ± 1.0	6.2 ± 1.3	0.32		
Albumin, mean ± SD, g/dL	3.6 ± 0.9	3.2 ± 0.6	0.08		
d-dimer, below: above reference value, n (%)	79 (66): 40 (34)	3 (38): 5 (42)	0.10		
Blood biochemistry on POD 3					
Hemoglobin, mean ± SD, g/dL	10.4 ± 2.0	9.2 ± 1.6	0.20		
Total protein, mean ± SD, g/dL	5.5 ± 0.8	4.9 ± 0.9	0.11		
Albumin, mean ± SD, g/dL	2.9 ± 0.6	2.4 ± 0.3	**0.05**	0.84	0.52 (0.01–462.47)

## Discussion

This is the first prospective study of VTE in UC surgery in Asia. The prevalence of perioperative VTE was 11.1% (15/134 patients) and no fatalities occurred. This prevalence is slightly higher than that reported in Western countries (2.7%–6.7%).^7–9^ Our study prospectively examined both symptomatic and asymptomatic VTEs. The incidence of VTE in patients with colorectal cancer is reported to be 2.75%–8.9%.^15, 24, 25^ The risk in patients with UC is not lower than that of patients with colorectal cancer. In Asia, VTE-conscious perioperative management is still as necessary as in Western countries.

Preoperative screening revealed VTE in 7 out of the 134 patients (5.2%), all of whom were asymptomatic. Forty-seven percent of VTE cases had already developed preoperatively. Therefore, preoperative VTE screening is important.

A hospital stay of over 5 days before surgery was a significant risk factor for preoperative VTE in the multivariate analysis. Severe inflammation was seen in 26.9% of patients; 18.8% of patients received corticosteroids over 20 mg per day, and 17.2% of patients had an insertion of a central venous catheter. However, none of these were significant risk factors for developing VTE. This may be because preoperative hospitalization of more than 5 days was a strong risk factor. Multiple risk factors are involved in the development of preoperative VTEs. Preoperative hospitalization for more than 5 days is considered to indirectly mean the need for strong medical treatment. The preoperative albumin level was maintained at 3.6 ± 0.9 g/dL, suggesting that the preoperative nutritional status was maintained appropriately. Age at surgery and diagnosis of cancer are well-known risk factors for VTE. However, these were not significant. The average age at the time of surgery in our study was 45 years old, which is a relatively young age. Thirty-five cases were indicated for surgery due to colitis-associated cancer. However, only 6 out of these 35 patients had advanced cancer. The low rate of advanced cancer was also considered to explain why this was not related to the prevalence of VTE. In the future, it is expected that age will become one of the risk factors due to an increase in the number of cases of surgery in older patients. Patients hospitalized preoperatively and requiring medical treatment are already considered to be at high risk for VTE.

Postoperative DVT occurred in 8 out of 127 patients (6.4%) and no deaths occurred. Eighty-five percent of VTEs occurred after POD 14. Only perioperative blood transfusion was a significant risk factor for postoperative VTE in the multivariate analysis.

The development of postoperative VTE is associated with various factors, including preoperative anemia, malnutrition, intraoperative hemorrhage, decreased circulating plasma volume, and dehydration.^7–9^ It is speculated that changes in portal blood flow and a decrease in extracellular fluid volume due to a dramatic decrease in water absorption in the digestive tract after total colectomy may be associated with this risk. Therefore, the risk of VTE should be considered in conditions that require perioperative transfusion.

Our findings show that it is important to perform a preoperative VTE screening and monitor perioperative volume management. These steps may help prevent fatal VTE.

Chemoprophylaxis use was not a significant risk factor. Postoperative chemoprophylaxis was performed only in 13% of patients. Chemoprophylaxis after surgery should be judged based on the risk of adverse events such as bleeding, because surgery for UC involves a wide range of resected tissue. On the other hand, multiple cases were diagnosed after 14 days. Therefore, the administration of chemoprophylaxis after initiation of oral feeds in patients with reduced bleeding risk may be considered.

VTE has been reported to contribute to readmission within 30 days after surgery.^26, 27^ Early postoperative dehydration is thought to be involved. However, in this study, the observation period ended at the discharge date, and the subsequent course could not be examined. Extended, long-term (4 weeks) prophylaxis has been demonstrated to decrease DVT risk from 9.7% to 0% in a randomized control trial of laparoscopic resection for colorectal cancer.^28^ Therefore, the efficacy of extended chemoprophylaxis early after discharge warrants further research.

Recently, the rate of laparoscopic surgery has increased. However, the proportion of laparoscopic surgery in this study was still small. Therefore, it is impossible to examine whether laparoscopic surgery is a risk factor for developing VTE. A single-center prospective study of laparoscopic surgery in 71 patients, including 35 cases of colectomy, found asymptomatic VTE in 18.3% of patients.^29^ The incidence and risk factors for postoperative VTE after laparoscopic surgery for UC are unknown and should be investigated in the future.

Highly effective nonsteroid medical therapies have been developed for UC treatments. Because there has been no major change in the selection policy of medical treatment after the case enrollment period of this study, the findings obtained in this study are valid even at present.

## Conclusion

The prevalence of perioperative VTE in Japan was 11.1% (15 out of 134 patients), which was similar to that reported in Western countries. Forty-seven percent of VTE cases had already developed preoperatively. Therefore, preoperative VTE screening is important. VTE-conscious perioperative management should be implemented in Asia as it is in Western countries.

## Data Availability

No new data were created or analyzed in the production of this manuscript.
